# Synergistic Redox Modulation for High‐Performance Nickel Oxide‐Based Inverted Perovskite Solar Modules

**DOI:** 10.1002/advs.202309111

**Published:** 2024-03-19

**Authors:** Yan Liu, Bin Ding, Gao Zhang, Xintong Ma, Yao Wang, Xin Zhang, Lirong Zeng, Mohammad Khaja Nazeeruddin, Guanjun Yang, Bo Chen

**Affiliations:** ^1^ State Key Laboratory for Mechanical Behavior of Materials Xi'an Jiaotong University Xi'an Shaanxi 710049 P. R. China; ^2^ Group for Molecular Engineering of Functional Materials Institute of Chemical Sciences and Engineering EPFL VALAIS Sion 1950 Switzerland

**Keywords:** interface reaction, nickel oxide, perovskite solar modules, redox modulation, synergistic

## Abstract

Nickel oxide (NiO_x_)‐based inverted perovskite solar cells stand as promising candidates for advancing perovskite photovoltaics towards commercialization, leveraging their remarkable stability, scalability, and cost‐effectiveness. However, the interfacial redox reaction between high‐valence Ni^4+^ and perovskite, alongside the facile conversion of iodide in perovskite into I_2_, significantly deteriorates the performance and reproducibility of NiO_x_‐based perovskite photovoltaics. Here, potassium borohydride (KBH_4_) is introduced as a dual‐action reductant, which effectively avoids the Ni^4+^/perovskite interface reaction and mitigates the iodide‐to‐I_2_ oxidation within perovskite film. This synergistic redox modulation significantly suppresses nonradiative recombination and increases the carrier lifetime. As a result, an impressive power conversion efficiency of 24.17% for NiO_x_‐based perovskite solar cells is achieved, and a record efficiency of 20.2% for NiO_x_‐based perovskite solar modules fabricated under ambient conditions. Notably, when evaluated using the ISOS‐L‐2 standard protocol, the module retains 94% of its initial efficiency after 2000 h of continuous illumination under maximum power point at 65 °C in ambient air.

## Graphical abstract

The dual‐action reductant KBH_4_ is employed to suppress the harmful reaction between NiO_x_ and perovskite while simultaneously avoiding iodide oxidation in perovskite. High‐quality perovskite film with low‐defect density on NiO_x_@KBH_4_ is achieved during the deposition in ambient conditions. This significantly improves the power conversion efficiency and stability of perovskite solar modules.

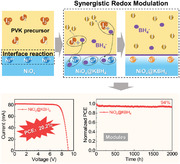

## 1 Introduction

Inverted perovskite solar cells (PSCs) hold significant promise for future commercialization of perovskite photovoltaics owing to the simple fabrication process, reliable operation, and compatibility with diverse perovskite‐based tandem device architectures.^[^ ^1^, ^2^, ^3^, ^4^, ^5^ ^]^ While higher power conversion efficiency (PCE) of inverted PSCs has been achieved using organic hole transport layers (HTLs) or self‐assembled monolayers (SAMs),^[^ ^5^, ^6^, ^7^, ^8^ ^]^ nickel oxide (NiO_x_) stands out as an outstanding HTL due to its cost‐effectiveness, excellent wetting properties, and ease large‐area manufacturing.^[^ ^9^, ^10^, ^11^, ^12^, ^13^, ^14^, ^15^ ^]^ However, the surface chemistry of NiO_x_ is complicated, involving different states of Ni species, including Ni^4+^, Ni^3+^, and Ni^2+^.^[^ ^16^, ^17^, ^18^, ^19^ ^]^ Among them, detrimental reactions between Ni^4+^ and A‐site organic cations of ABX_3_ structure of perovskite result in the formation of A‐site‐defective defects at the interface of perovskite film,^[^ ^20^ ^]^ which leads to PCEs of PSCs on HTL of NiO_x_ alone lagging behind those using organic HTLs and SAMs. Moreover, the iodide ions present in perovskite precursor are vulnerable to oxidation into I_2_ (or $I_{3}^{-}$) during the storage and utilization of precursor solutions, which significantly compromises the performance and reproducibility of PSCs.^[^ ^21^, ^22^ ^]^ This concern is amplified when dealing with large‐area perovskite modules, as they are often manufactured in ambient conditions.^[^ ^23^, ^24^ ^]^

To address this issue, recent research efforts have explored various strategies, primarily focused on interface modification and the incorporation of reducing agents into perovskite precursors.^[^ ^25^, ^26^, ^27^, ^28^, ^29^, ^30^, ^31^, ^32^, ^33^, ^34^, ^35^, ^36^, ^37^, ^38^ ^]^ Effective approaches to interface modification involve the reduction of Ni^4+^ on the NiO_x_ surface^[^ ^25^, ^26^ ^]^ and the introduction of a physical separation layer,^[^ ^27^, ^28^ ^]^ both of which have proven their ability to suppress the detrimental redox reactions between the perovskite film and NiO_x_ layer. However, the implementation of physical separation layers, such as SAMs^[^ ^4^, ^39^ ^]^ and PTAA^[^ ^29^ ^]^ increases the contact angle of perovskite precursor on HTLs, potentially posing challenges for the upscaling of large‐area perovskite film deposition;^[^ ^40^, ^41^ ^]^ meanwhile, the atomic layer deposition of Al_2_O_3_ as physical separation layer adds cost and complexity to the device fabrication.^[^ ^30^ ^]^ In response to the issue of I^−^ oxidation, recent developments have introduced reducing agents, such as benzylhydrazine hydrochloride and 3‐hydrazinobenzoic acid, into the perovskite precursor solution.^[^ ^37^, ^38^ ^]^ Nevertheless, to date, a comprehensive strategy that simultaneously overcomes the redox reaction issue at the NiO_x_/perovskite interface and the problem of I^−^ oxidation within the perovskite precursor remains relatively underexplored.

In this work, we propose for the first time a synergistic redox modulation strategy utilizing potassium borohydrides (KBH_4_) to simultaneously eliminate the detrimental Ni^4+^ at the surface of NiO_x_ HTL and suppress the iodide‐to‐I_2_ oxidation within perovskite film. The surface treatment of NiO_x_ HTL by KBH_4_ can reduce the high‐valent Ni^4+^ species on the NiO_x_ surface through its strongly reducing borohydride groups, and the residual KBH_4_ on HTL can further reduce the oxidized I_2_ within the perovskite precursor and suppress the generation of I_2_ within the perovskite film during operation. As a result, the planar NiO_x_‐based inverted PSCs obtain a champion PCE of 24.17%; and the perovskite mini‐module (with an aperture area of 29.0 cm^2^) achieves a record efficiency of 20.2%, accompanied by significantly enhanced operational stability of *T*_90_ lifetime over 2000 h at maximum power point (MPP) under continuous light illumination at 65 °C in ambient air.

## 2 Results and Discussion

### 2.1 Reduction of NiO_x_ Surface

**Figure 1:**
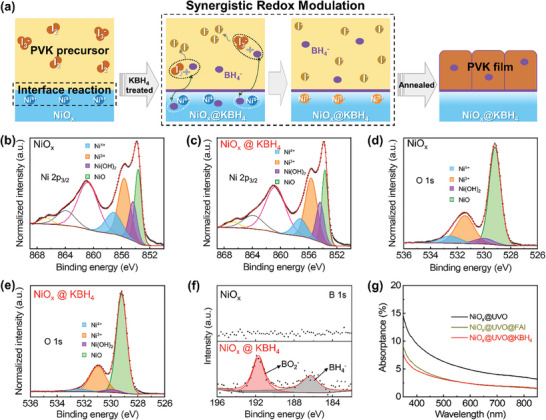
The mechanism of reduction reaction on NiO_x_ surface. a) Illustration of the influence and mechanism of KBH_4_ treatment for deposition of perovskite film on NiO_x_. XPS spectra of Ni 2p_3/2_ for b) bare NiO_x_ and c) NiO_x_@KBH_4_. XPS spectra of O 1s for d) bare NiO_x_ and e) NiO_x_@KBH_4_. All treatments were followed by a DMF wash. f) XPS spectra of B 1s of bare NiO_x_ and NiO_x_@KBH_4_ films. g) Absorptance spectra of UVO‐treated NiO_x_ film before and after 3‐mins soaking with FAI or KBH_4_ solution in DMF. Both treatments were followed by a DMF wash before absorptance measurement.

**Figure** **1**a illustrates how KBH_4_ can effectively eliminate adverse reactions at the NiO_x_/perovskite interface while simultaneously reducing the I_2_ content within the perovskite precursor. Our investigation first focused on whether the reductant KBH_4_ applied to NiO_x_ denoted as NiO_x_@KBH_4_, could suppress the presence of harmful Ni^4+^ species on the surface of the NiO_x_ HTL. To gain insights into the change in surface species on the NiO_x_ surface resulting from redox processes, X‐ray photoemission spectroscopy (XPS) analyses were performed. The Ni 2p_3/2_ and O 1s XPS data were deconvoluted following established procedures outlined in the literature.^[^ ^20^, ^26^, ^39^ ^]^ As shown in Figure 1b,c and Figure S1 and Table S1 (Supporting Information), the XPS peaks located at ≈855.6 and ≈857.1 eV correspond to Ni^3+^ and Ni^4+^, respectively. After spin coating KBH_4_ onto NiO_x_ HTL, the detected Ni^4+^ content via Ni 2p_3/2_ XPS was reduced, accompanied by an increase in Ni^3+^. Notably, the XPS data of O 1s consistently demonstrate a noticeable reduction in the amount of Ni^4+^ following the KBH_4_ treatment (Figure 1d,e), aligning with the results obtained from the XPS analysis of Ni 2p_3/2_.

To further confirm the vigorous reaction between Ni^4+^ and KBH_4_ solution, we directly added NiO_2_ powder into the KBH_4_ solution, resulting in the generation of abundant bubbles, which should be hydrogen,^[^ ^42^, ^43^ ^]^ as demonstrated in Video S1 (Supporting Information). Moreover, based on the previous study,^[^ ^43^ ^]^ the B 1s spectra peaks at ≈192 and ≈187 eV in Figure 1f reveal the present of ${\mathrm{BO}}_{2}^{-}$ and ${\mathrm{BH}}_{4}^{-}$, respectively. Therefore, we speculate that the reaction occurring on the NiO_x_ surface may potentially be elucidated through the following equation^[^ ^42^, ^43^ ^]^:

(1) $$4\mathrm{Ni}O_{2}+{\mathrm{BH}}_{4}^{-}\to 2Ni_{2}O_{3}+{\mathrm{BO}}_{2}^{-}+2H_{2}↑$$

The interaction between the surface Ni^4+^ species on NiO_x_ with A‐site organic cations or the reductant is further confirmed. To amplify the presence of Ni^4+^ on the surface and facilitate the differentiation of redox reactions between Ni^4+^ and FAI or KBH_4_, we applied UV–ozone (UVO) surface treatment, a known method to increase the concentration of Ni^4+^ on the NiO_x_ surface,^[^ ^20^ ^]^ transforming the formerly transparent NiO_x_ film into a semi‐transparent film with a light black color (Figure S2, Supporting Information). After rinsing the UVO‐treated NiO_x_ film with either FAI solution or KBH_4_ solution, a distinct bleaching effect that restored the film's transparency was observed, as evidenced by photographs in Figure S2 (Supporting Information) and changes in absorption spectra in Figure 1g. This observation validates the interaction of FAI with surface Ni^4+^ on NiO_x_@UVO, consistent with previous research findings.^[^ ^36^ ^]^ Additionally, it underscores the capacity of KBH_4_ to effectively reduce surface Ni^4+^ on NiO_x_@UVO, thereby preventing the detrimental reaction between NiO_x_ and the perovskite during subsequent perovskite deposition. Notably, the Ni^4+^ content in NiO_x_@KBH_4_ and NiO_x_@KBH_4_@FAI were nearly identical (Figure S3 and Table S2, Supporting Information). This observation underscores the effectiveness of KBH_4_ in preventing the detrimental reaction between Ni^4+^ and FAI.

### 2.2 Iodine Reduction by NiO_x_@KBH_4_

XPS and scanning electron microscopy with energy dispersive X‐ray spectroscopy (SEM/EDX) analysis reveal the presence of ${\mathrm{BH}}_{4}^{-}$ and K^+^ from the surface of KBH_4_ treated NiO_x_ (Figure 1f; Figure S4,S5, Supporting Information), suggesting that some KBH_4_ remain on surface after their interaction with NiO_x_. These residual KBH_4_ may act as a pre‐buried reductant, potentially influencing the subsequent perovskite deposition.

**Figure 2:**
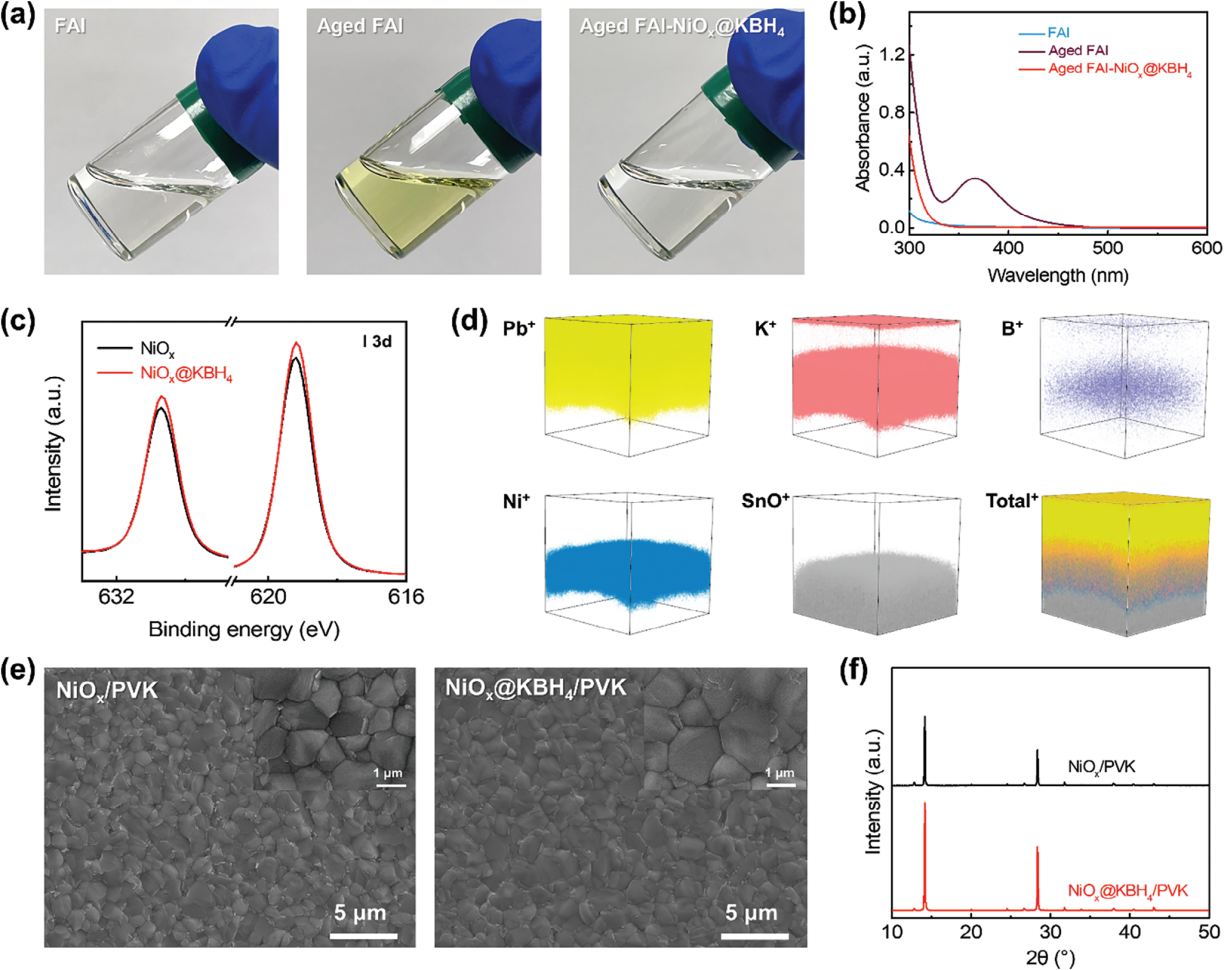
Impact of NiO_x_@KBH_4_ on iodine reduction. a) Photographs and b) UV–vis absorption spectra of the pristine FAI solution, after aged in ambient air for 3 days, and the 3‐day‐aged FAI solution treated with NiO_x_@KBH_4_ substrate. c) XPS spectra of I 3d for the perovskite films prepared on bare NiO_x_ and NiO_x_@KBH_4_. d) TOF‐SIMS profile of glass/FTO/NiO_x_@KBH_4_/perovskite. e) Top‐view scanning electron microscope (SEM) images and f) XRD patterns of the perovskite films on bare NiO_x_ and NiO_x_@KBH_4_.

As shown in **Figure** **2**a, after being exposed to ambient air for 3 days, the color of the FAI in DMF solution turned light yellow, accompanied by the emergence of an absorption peak at ≈365 nm in the solution's absorption spectrum (Figure 2b). This peak can be attributed to the presence of $I_{3}^{-}$, which forms through the combination of I_2_ and I^−^ ions.^[^ ^38^ ^]^ Notably, even when the freshly prepared FAI solution is briefly exposed to ambient air for a short duration of 1 min during the coating process, I^−^ undergoes oxidation, yielding a noticeable $I_{3}^{-}$ absorption peak (Figure S6, Supporting Information). Subsequently, we dropped the 3‐day‐aged FAI solution onto NiO_x_@KBH_4_ substrates and immediately collected back the solution for analysis. A remarkable transformation was observed: the solution's color changed back to transparent (Figure 2a) and the absorption peak of $I_{3}^{-}$ almost entirely disappeared from the absorption spectrum (Figure 2b). These findings reveal the effective capability of NiO_x_@KBH_4_ in reducing the presence I_2_/$I_{3}^{-}$ species within the perovskite solution.

The XPS spectra revealed an increased peak area in the I 3d region of the perovskite film deposited on the NiO_x_@KBH_4_ substrate compared to the bare NiO_x_ substrate (Figure 2c). In contrast, the Pb 4f peak remained nearly constant (Figure S7, Supporting Information). This observation suggests an elevated I:Pb ratio within the perovskite film deposited on NiO_x_@KBH_4_ due to the reduction of $I_{3}^{-}$, which effectively prevents the sublimation of I_2_.^[^ ^44^ ^]^ Besides accumulation on top of NiO_x_ HTL, the substantial amount of K^+^ and B^+^ within the perovskite layer (Figure 2d) was detected by time‐of‐flight secondary ion mass spectrometry (TOF‐SIMS), which confirms the interaction between pre‐buried KBH_4_ on HTL and perovskite film. In addition to the reduction effect of ${\mathrm{BH}}_{4}^{-}$, K^+^ ions might complex with I^−^ into benign compounds at the grain boundaries and surfaces, thereby suppressing the migration and potential oxidation of iodide.^[^ ^45^ ^]^ Perovskite films on both NiO_x_@KBH_4_ and bare NiO_x_ substrates exhibited uniform and pinhole‐free morphology with intimate contact with the substrates (Figure 2e; Figure S8, Supporting Information), which may be due to the good wettability of perovskite precursor on both NiO_x_ and NiO_x_@KBH_4_ substrates compared to NiO_x_@MeO‐2PACZ and NiO_x_@PTAA (Figure S9, Supporting Information).^[^ ^40^, ^41^, ^46^ ^]^ The grain size of the film deposited on NiO_x_@KBH_4_ substrates is slightly larger than that deposited on bare NiO_x_ substrates, as shown in Figure S10 (Supporting Information). This phenomenon of increased grain size was also observed in other reduction strategies.^[^ ^21^, ^22^ ^]^ X‐ray diffraction (XRD) analysis in Figure 2f shows that there is minimal disparity in the crystallinity of perovskite films between deposition on the NiO_x_@KBH_4_ and bare NiO_x_ substrates.

### 2.3 Optoelectronic and Trap State Characterization

**Figure 3:**
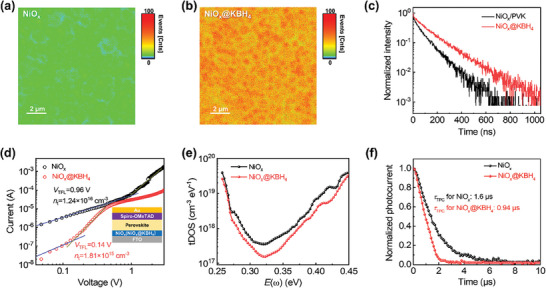
Analysis of charge extraction and trap state density. Steady PL mapping of perovskite films deposited on a) NiO_x_ and b) NiO_x_@KBH_4_. All perovskite films were excited by a 450 nm light source from the perovskite side. c) TRPL spectra for perovskite films deposited on NiO_x_ and NiO_x_@KBH_4_ with excitation from the perovskite side. d) SCLC measurements of hole‐only devices based on NiO_x_ and NiO_x_@KBH_4_ as bottom HTL. e) tDOS and f) normalized TPC curves for PSCs based on NiO_x_ and NiO_x_@KBH_4_.

In order to evaluate the optoelectronic characteristics of the perovskite thin films, photoluminescence (PL) mapping and time‐resolved photoluminescence (TRPL) spectra were employed. Although the perovskite films deposited on bare NiO_x_ and NiO_x_@KBH_4_ in ambient conditions both exhibited a relatively uniform distribution on PL mapping (**Figure** **3**a,b). It was evident that the perovskite film prepared on NiO_x_@KBH_4_ showed a notably stronger PL emission compared to the control film on bare NiO_x_ when excitation from either the perovskite film side or glass substrate side (Figure S11, Supporting Information). Furthermore, the TRPL spectra indicated that the decay lifetime of the perovskite film on NiO_x_@KBH_4_ substrate (132.41 ns) surpassed that of the control film on bare NiO_x_ substrate (82.16 ns) when excitation from the perovskite film side (Figure 3c and Figure S12, Supporting Information). When the TRPL measurement was excited from the glass side, TRPL spectra demonstrated two exponent decay properties as shown in Figure S13 (Supporting Information). According to prior studies,^[^ ^39^, ^47^, ^48^ ^]^ the fast decay (τ_1_) is associated with charge‐carrier separation, revealing the quenching process. However, we observed that the τ_1_ values for NiO_x_ and NiO_x_@KBH_4_ were similar, measuring 1.14 and 1.37 ns, respectively. This similarity suggests that KBH_4_ does not enhance carrier extraction. We hypothesize that the comparable carrier extraction might be attributed to minimal changes in the valence band maximum (VBM) between NiO_x_ and NiO_x_@KBH_4_, as verified by UPS measurements in Figure S14 (Supporting Information). A longer decay time (τ_2_) was observed for the perovskite film deposited on NiO_x_@KBH_4_, indicating improved perovskite film quality. These PL and TRPL results reveal that there is a reduction in non‐radiative recombination for perovskite film prepared on NiO_x_@KBH_4_.

To quantitatively assess the trap densities of these perovskite films, the hole‐only devices were fabricated with the configuration of glass/FTO/NiO_x_ (or NiO_x_@KBH_4_)/perovskite/Spiro‐oMeTAD/Ag. The dark current–voltage (*I*–*V*) characteristics of these devices are presented in Figure 3d. The specific trap‐state densities were determined by fitting *I*–*V* curves using the Mott‐Gurney law.^[^ ^49^, ^50^ ^]^ The perovskite film prepared on NiO_x_@KBH_4_ exhibited a lower trap‐filled limit voltage (*V*_TFL_) value of 0.14 V and a reduced trap density of 1.81 × 10^15^ cm^−3^, in contrast to *V*_TFL_ of 0.96 V and trap density of 1.24 × 10^16^ cm^−3^ for the film prepared on bare NiO_x_. The reduced trap density was further confirmed by the results of thermal admittance spectroscopy measurement. As shown in Figure 3e, the PSC employing the NiO_x_@KBH_4_ HTL displayed a lower trap density of states (tDOS) than the device on bare NiO_x_. The trap density at the region from 0.25 to 0.32 eV is attributed to $I_{3}^{-}$ and the trap density at the region from 0.32 to 0.45 eV is attributed to $I_{i}^{+}$.^[^ ^23^ ^]^ Therefore, tDOS result reveals that KBH_4_ treatment suppresses the harmful oxidation of I^−^ into $I_{3}^{-}$ and $I_{i}^{+}$. Furthermore, the reduction in non‐radiative recombination losses due to reduced trap densities was corroborated by the transient photocurrent (TPC) measurements. The decay time of TPC curves (Figure 3f) notably decreases from 1.6 to 0.94 µs after KBH_4_ modification, confirming the suppressed carrier recombination within the device based on NiO_x_@KBH_4_.

### 2.4 Performance of Perovskite Solar Cells and Modules

**Figure 4:**
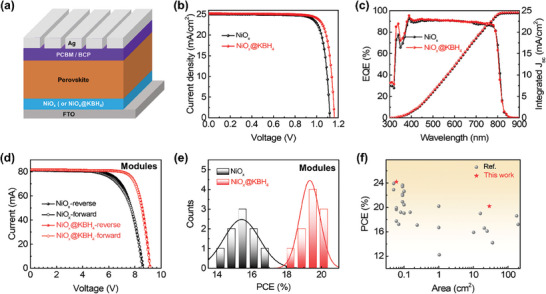
Performance of the perovskite solar cells and modules. a) Architecture of NiO_x_‐based inverted PSCs. b) *J–V* curves and c) EQE spectra and integrated *J*_SC_ curves of PSCs employing NiO_x_ or NiO_x_@KBH_4_ HTL. d) *I–V* curves of the champion perovskite solar modules using NiO_x_ or NiO_x_@KBH_4_ HTL with an aperture area of 29 cm^2^. e) Distribution of PCE derived from 10 modules of each device type. f) A summary of PCE versus area for our NiO_x_‐based perovskite solar devices and reported devices on HTL of NiO_x_ with details in Tables S4,S5 (Supporting Information).

Having demonstrated the enhanced carrier lifetime through the NiO_x_ modification with KBH_4_, we proceeded to evaluate the performance of the corresponding perovskite photovoltaic devices with a p‐i‐n structure consisting of glass/FTO/NiO_x_ (or NiO_x_@KBH_4_)/perovskite/PCBM/BCP/Ag (**Figure** **4**a). The perovskite films were deposited in ambient conditions. The *J*–*V* characteristics of the best‐performing PSCs of each condition are shown in Figure 4b. Notably, the KBH_4_ modification resulted in an increase in the open‐circuit voltage (*V*_OC_) from 1.122 to 1.164 V, accompanied by an improvement in the fill factor (FF) from 81.6% to 82.4%, thus improving the PCE of the PSCs from 23.13% to 24.17% (Figure 4b), which was confirmed by the stabilized power output in Figure S15 (Supporting Information). The integrated short‐circuit current density (*J*_SC_) from the external quantum efficiency (EQE) curve matched well with the *J*_SC_ measured under the solar simulator (Figure 4c). Moreover, the average PCE of PSCs prepared on NiO_x_@KBH_4_ increased from 22.42% to 23.80%, displaying a notably confined distribution of photovoltaic parameters (Figure S16, Supporting Information). This indicates that the dual‐function of KBH_4_ modification improves the PCE and reproducibility of PSCs. Furthermore, for the perovskite solar modules (aperture area of 29.0 cm^2^) with the same configuration as the small‐area PSCs, a champion PCE of 20.2% was attained using the NiO_x_@KBH_4_ HTL (Figure 4d; Table S3, Supporting Information). No hysteresis was observed on perovskite solar modules with NiO_x_@KBH_4_ HTL (Figure 4d). The average PCE of modules was increased from 15.42% to 19.34% after KBH_4_ modification (Figure 4e). This achievement represents the record efficiency of perovskite solar modules employing a NiO_x_ HTL, supported by the latest device efficiency data shown in Figure 4f and Tables S4,S5 (Supporting Information).

**Figure 5:**
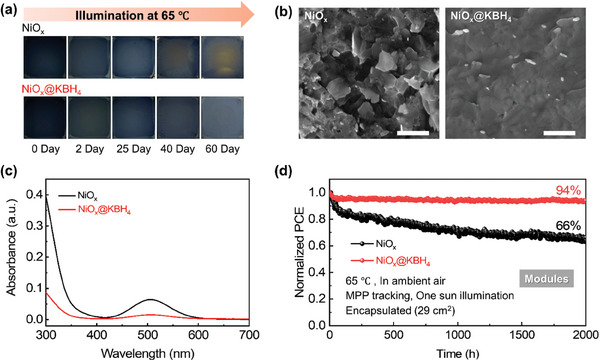
Stability of the perovskite films and modules. a) The photographs of unencapsulated perovskite films deposited on bare NiO_x_ and NiO_x_@KBH_4_ under one sun illumination of 100 mW/cm^2^ at 65 °C in an N_2_ atmosphere. b) SEM images of perovskite film bottom surface that peeled off from NiO_x_ and NiO_x_@KBH_4_ substrates after light‐soaking under one sun for 60 days at the temperature of 65 °C. Scale bars, 1 µm. c) Absorption spectra of the toluene solution, in which perovskite films deposited on bare NiO_x_ (black) and NiO_x_@KBH_4_ (red) were immersed, after one sun illumination for 10 h. d) MPP tracking of encapsulated perovskite solar modules under one sun illumination at 65 °C in ambient air.

In addition to evaluating PCE, we subjected the perovskite films on NiO_x_@KBH_4_ to long‐term stability testing to assess the impact of KBH_4_ modification. The color change of unencapsulated perovskite films under illumination at 65 °C in an N_2_ atmosphere is visually captured in **Figure** **5**a. The degradation of perovskite film deposited on bare NiO_x_ was observed to be more rapid compared to that on NiO_x_@KBH_4_ (Figure 5a), which is further affirmed by the corresponding SEM image in Figure 5b and XRD results in Figure S17 (Supporting Information). To quantify the generation of I_2_ within the films during light soaking, we immersed perovskite films into the toluene solution and then applied light soaking. After 10 h of continuous one sun light soaking, the absorption spectra revealed a significantly reduced amount of I_2_ (located at ≈500 nm) generated from the films on NiO_x_@KBH_4_ compared to that on bare NiO_x_ (Figure 5c). Remarkably, considering that NiO_x_@KBH_4_ retains its reducing capability even after multiple time of coating with aged FAI solution (Figure S18, Supporting Information), it is suggested that residual KBH_4_ left within the device after perovskite film deposition. This residual KBH_4_ serves as a reductant agent to suppress the generation of I_2_ within the perovskite films during operation thereby enhancing the stability of the films under illumination. We aged the encapsulated perovskite solar modules at MPP tracking in ambient air under one sun illumination at 65 °C, according to ISOS‐L‐2 standard protocol.^[^ ^51^ ^]^ The operational stability of perovskite modules based on bare NiO_x_ and NiO_x_@KBH_4_ is compared in Figure 5d. After 2000 h of continuous light‐soaking, the NiO_x_@KBH_4_‐based module maintained 94% of its initial PCE, whereas the NiO_x_‐based module exhibited significantly poorer operational stability with only 66% of the initial PCE remaining.

## 3 Conclusion

In summary, this study presents a synergistic redox modulation strategy employing KBH_4_, which accomplishes the dual objectives of eliminating the detrimental Ni^4+^ on the surface of NiO_x_ HTL and reducing I_2_ content within perovskite film. The mechanism of interaction between the harmful high‐valent Ni^4+^ and KBH_4_ was comprehensively elucidated. The KBH_4_ on NiO_x_ HTL not only can reduce the $I_{3}^{-}$ in perovskite precursor, but also suppress the generation of I_2_ during the operation of PSCs. This effect of synergistic redox modulation subsequently reduced the density of non‐radiative recombination centers, increased the carrier lifetime, and improved device stability. Notably, the multifunctional additive KBH_4_ enabled the NiO_x_‐based PSCs and perovskite solar module with record PCE of 24.17% and 20.2%, respectively. Furthermore, KBH_4_ modification also showed its influence on the long‐term stability of perovskite solar modules, with a retention of 94% of the initial PCE after 2000 h of continuous illumination at 65 °C in ambient air. This study introduces a novel redox modulation approach that holds the potential to advance the fabrication of high‐efficiency NiO_x_‐based perovskite solar devices.

## 4 Experimental Section

### Materials

Nickel(II) acetylacetonate (95%), cesium chloride (CsCl, 99%), acetonitrile (99.8%), ethanol (99.5%), dimethylformamide (DMF, 99.8%), dimethyl sulfoxide (DMSO, 99.9%), chlorobenzene (99.8%), isopropanol (99.5%), and bathocuproine (BCP, 99.99%) were all purchased from Sigma‐Aldrich. Potassium borohydride (KBH_4_, 98%) was purchased from Alfa Aesar. Lead iodide (PbI_2_, 99.99%) was purchased from TCI Chemicals. Methylammonium iodide (MAI, 99.99%), methylammonium chloride (MACl, 99.99%), and formamidinium iodide (FAI, 99.99%) were purchased from Greatcell Solar Materials Pty Ltd. Phenyl‐C61‐butyric acid methyl ester (PCBM, 99.5%) was purchased from Luminescence technology corp., Taiwan. Silver (Ag, 99.99%) was purchased from Laiyan Technology Co., Ltd. All the materials were used as received.

### Perovskite Solar Cell Fabrication

FTO substrates (TEC 7–10 Ω, Nippon Sheet Glass Co., Japan) were ultrasonically cleaned by washing with soap water, acetone, ethanol, and deionized water for 20 mins, respectively. The NiO_x_ HTL was prepared using a spray pyrolysis method on the substrate.^[^ ^31^ ^]^ The mixture solution, acetonitrile and ethanol (with 95:5 volume ratio, 40 mL) of nickel acetylacetonate (0.02 mol L^−1^), was sprayed by an air nozzle with 0.3 mm caliber onto hot FTO glasses (500 °C) in ambient air. After spraying, the NiO_x_/FTO was further treated at 500 °C for another 30 mins and cooled naturally. Then, the potassium borohydride (KBH_4_, 5 mg mL^−1^ in ethanol) was then spin‐coated on NiO_x_ HTLs at 5000 rpm for 30 s. The Cs_0.05_MA_0.05_FA_0.9_PbI_3_ perovskite precursor solution (1.3 m) was prepared by adding the 659 mg PbI_2_, 10 mg MAI, 11 mg CsCl, and 212 mg FAI in a mixed solvent of DMF and DMSO (4:1 in volume), respectively. The filtered perovskite precursor solution was spin‐coated onto the NiO_x_ substrate at 2000 rpm for 10 s, and then transferred to a gas‐pump chamber with 5 Pa, pumping for 60 s. Then the mirror‐like dry film was annealed at 115 °C for 15 min. The deposition of perovskite films was carried out in ambient air with a relative humidity of 40–50%. Then, a PCBM solution (20 mg mL^−1^ in chlorobenzene) was spin‐coated at 2000 rpm for 30 s, and then annealed at 100 °C for 10 min. Subsequently, a very thin layer of BCP (0.5 mg mL^−1^ in isopropanol) was spin‐coated at 5000 rpm for 30 s on the top of the PCBM layer and annealed at 100 °C for 10 min. Finally, a 100 nm Ag was deposited via thermal evaporation under a high vacuum (<5 × 10^−4^ Pa). The device area of PSCs was 0.06 cm^2^.

### Perovskite Solar Module Fabrication

Perovskite solar modules, with 8 sub‐cells connected in series, were prepared on FTO glass substrates with a size of 6.5 cm × 7.0 cm. The series interconnection of the module was realized by P1, P2, and P3 lines, which were patterned using a laser scribing system with a 1064 nm and a power of 20 W (Trotec). The FTO substrates were pre‐patterned for P1 (a width of 40 µm) by an average laser power of 60% under a speed of 300 mm s^−1^ with a frequency of 65 kHz and pulse width of 120 ns. The subsequent deposition processes for NiO_x_, perovskite, PCBM, and BCP layers were the same as those for small‐sized PSCs. The P2 lines (a width of 100 µm) were patterned before the Ag evaporation process with an average laser power of 15% under a speed of 1000 mm s^−1^, frequency of 65 kHz, and pulse duration of 120 ns. After a 100 nm‐thick Ag layer was deposited, the P3 line (a width of 40 µm) was engraved under the same scribing condition as the P2 line. The width of the total dead area was ≈280 µm with a geometric fill factor (GFF) of ≈95.7%.

### Characterization

The ultraviolet‐visible spectra of the NiO_x_ and perovskite films were measured in an ultraviolet‐visible‐near infrared spectrophotometer (PE Lambda950). The X‐ray photoelectron spectroscopy (XPS) was performed in an X‐ray photoelectron spectrometer (Thermo Fisher ESCALAB Xi+), using 400 W monochrome Al Kα (1486.6 eV) radiation. Field‐emission scanning electron microscope (SEM, FEI VERIOS 460) was used to examine the surface and cross‐sectional morphology of perovskite films. The X‐ray diffraction patterns were obtained using an X‐ray diffractometer (Brucker D8 ADVANCE) with Cu Kα radiation at a scanning speed of 0.1° s^−1^. Depth profiling data was obtained with the TOF‐SIMS 5 system from ION‐TOF. The time‐resolved PL (TRPL) decay was measured in a steady‐state‐transient fluorescence spectrometer (Edinburgh FLS980) with the laser diode at the wavelength of 450 nm. The PL mapping was performed using a super‐resolution confocal microscope (Leica TCS SP8 STED 3X). The TPC measurements were measured by a homemade system, which converted the 20HZ continuous laser (ZL‐532‐500 mV) signal into a pulse signal with a wave converter (RIGOL DG812) and received it with an oscilloscope (Tektronix MDO3014). The *J*–*V* and *I*–*V* curves of the perovskite solar devices were measured with a 2400 series source meter, Keithley Instruments, under the illumination of the solar simulator (Newport, Class AAA), AM 1.5G filter (Sol3A, Oriel) at the light intensity of 100 mW cm^−2^, calibrated using a standard Si reference cell. The aperture area of the module under the *I* *–V* measurement was 29.0 cm^2^. The incident photo‐to‐current conversion efficiency (IPCE) spectra were obtained with a Qtest Station 1000ADX system (Growntech, Inc.) in the air without bias light. The stability of encapsulated perovskite solar modules was tested at the maximum power point (MPP) by using an MPP tracking algorithm under 100 mW cm^−2^ in ambient air at 65 °C.

## Conflict of Interest

The authors declare no conflict of interest.

## Supporting information

Supporting Information

Supplemental Video 1

## Data Availability

The data that support the findings of this study are available from the corresponding author upon reasonable request.
