# Supplementary material for: Synergistic Redox Modulation for High‐Performance Nickel Oxide‐Based Inverted Perovskite Solar Modules
Source: Adv Sci (Weinh). 2024 Mar 19;11(21):2309111. doi: 10.1002/advs.202309111 (PMC11151082; doi:10.1002/advs.202309111)
Supplement: Supplementary file 1 — Supporting Information [file ADVS-11-2309111-s001.pdf]

ADVANCED SCIENCE
Open Access

Supporting Information

for *Adv. Sci.*, DOI 10.1002/advs.202309111

Synergistic Redox Modulation for High-Performance Nickel Oxide-Based Inverted Perovskite Solar Modules

Yan Liu, Bin Ding, Gao Zhang, Xintong Ma, Yao Wang, Xin Zhang, Lirong Zeng, Mohammad Khaja Nazeeruddin, Guanjun Yang and Bo Chen**

Supporting Information

Synergistic Redox Modulation for High-Performance Nickel Oxide-Based Inverted Perovskite Solar Modules

Yan Liu,‡ Bin Ding,‡ Gao Zhang, Xintong Ma, Yao Wang, Xin Zhang, Lirong Zeng, Mohammad Khaja Nazeeruddin, Guanjun Yang, and Bo Chen**

Y. Liu, G. Zhang, X. Ma, Y. Wang, X. Zhang, L. Zeng, G. Yang, and B. Chen

State Key Laboratory for Mechanical Behavior of Materials, Xi'an Jiaotong University, Xi'an, Shaanxi 710049, P.R. China.

E-mail: ygj@mail.xjtu.edu.cn (G.Y.), bochen@xjtu.edu.cn (B.C.)

B. Ding, M. K. Nazeeruddin

Group for Molecular Engineering of Functional Materials, Institute of Chemical Sciences and Engineering, EPFL VALAIS, Sion 1950, Switzerland.

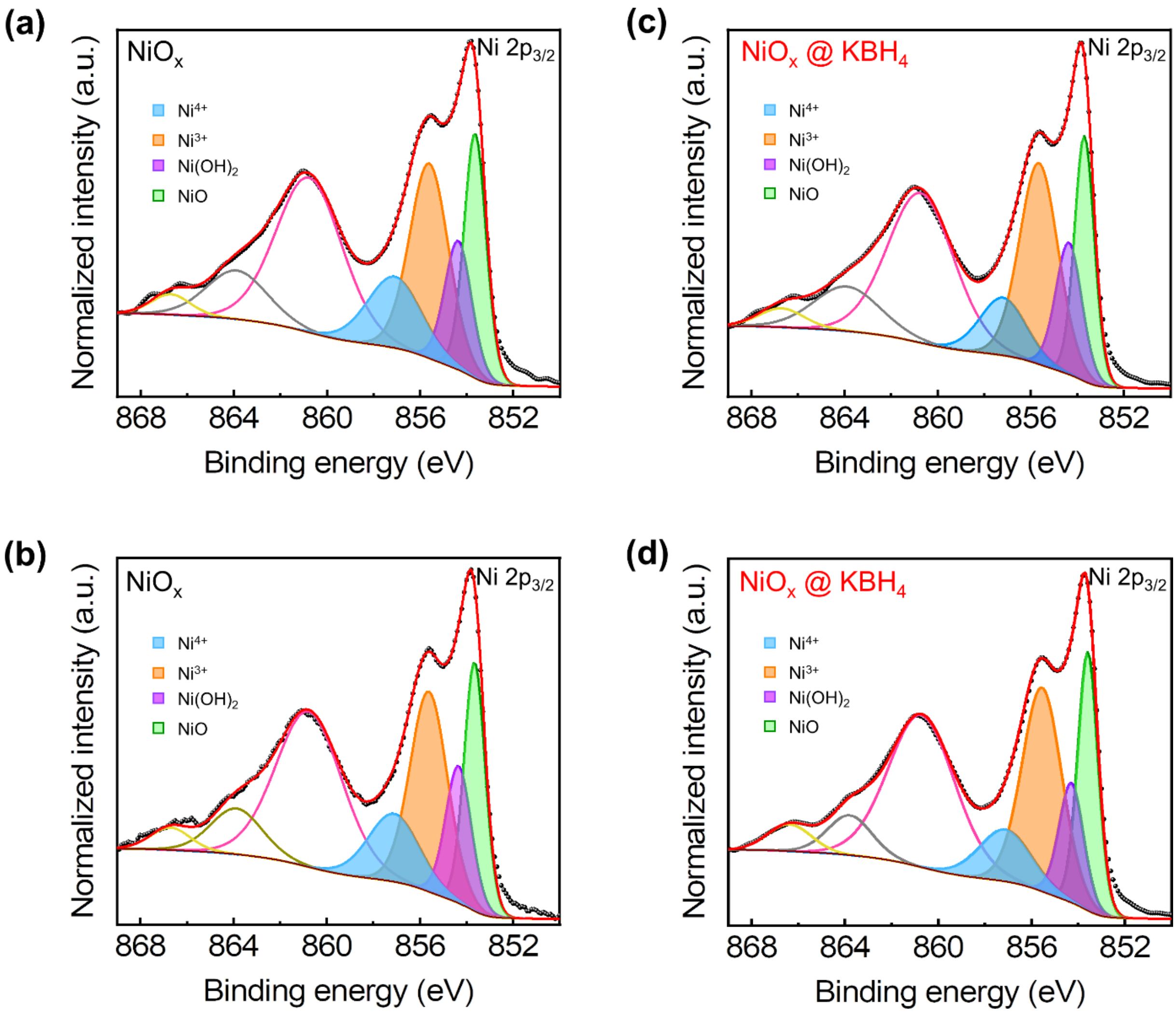

Figure S1. XPS spectra of Ni 2p3/2 of (**a, b**) bare NiO_x and (**c, d**) NiO_x@KBH_4.

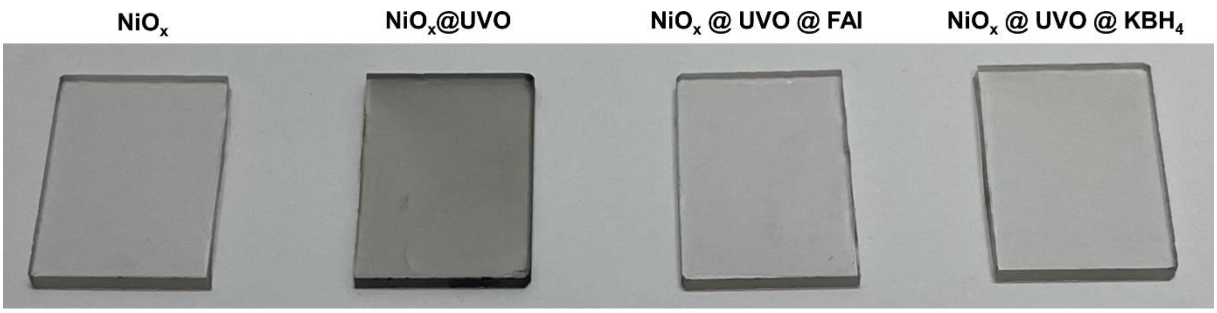

Figure S2. Photograph of different NiO_x films: as deposited, treated with UVO for 60 minutes, and treated with UVO followed by rinsing with FAI solution or KBH_4 solution in DMF for 3 minutes. All treatments were followed by a DMF wash.

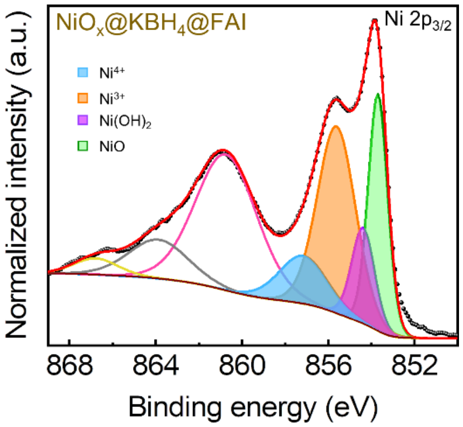

Figure S3. XPS spectra of Ni 2p3/2 of NiO_x@KBH_4@FAI.

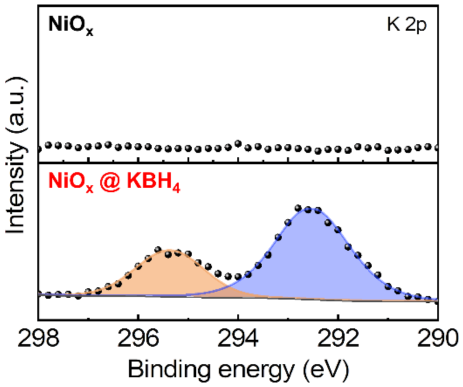

Figure S4. XPS spectra of K 2p analysis of bare NiO_x and NiO_x@KBH_4 films.

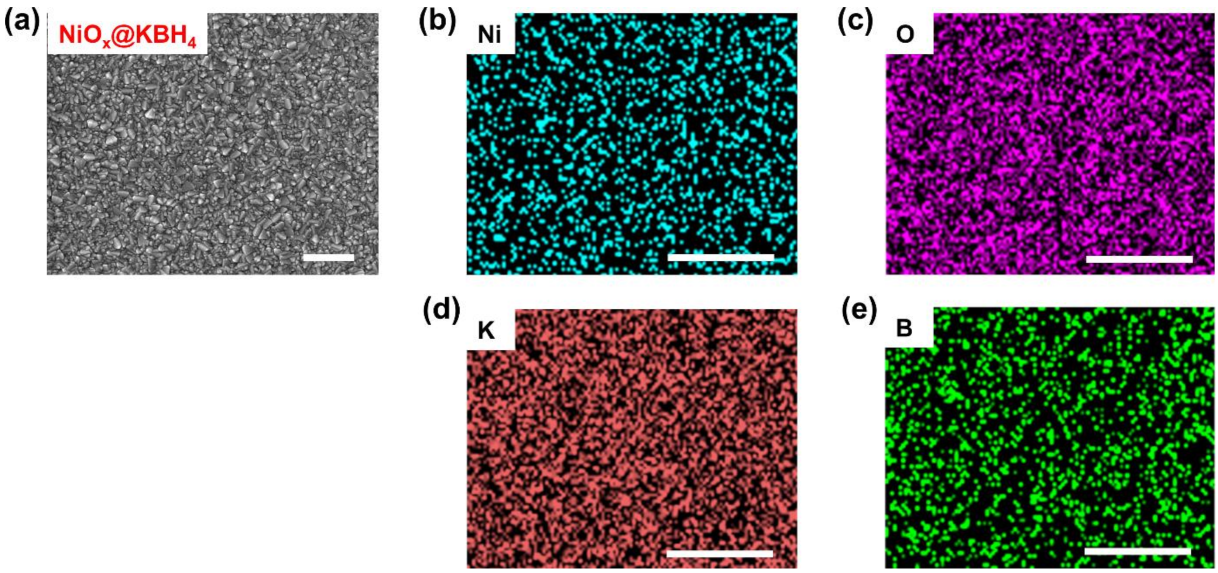

Figure S5. SEM-EDX analysis results of NiO_x@KBH_4 surface. (**a**) SEM image for EDX analysis. Scale bar, 2 µm. (**b-e**) EDX surface-scan analysis for Ni, O, K, and B, respectively. Scale bars, 2.5 µm.

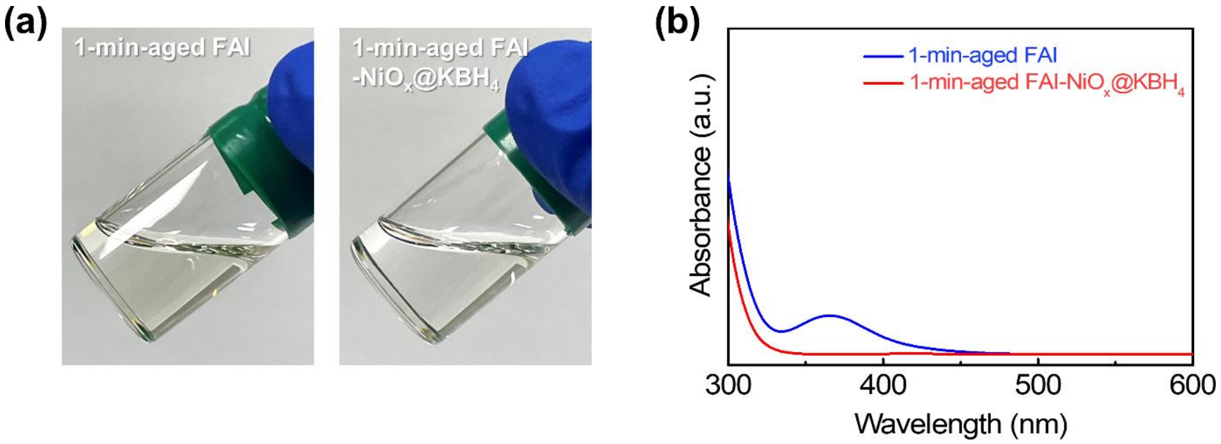

Figure S6. Interaction between 1-min-aged FAI solution and NiO_x@KBH_4. (**a**) Photographs and (**b**) UV-vis absorption spectra of the 1-min-aged FAI solution before and after treatment with NiO_x@KBH_4 substrates. 1-min-aged FAI solution was prepared by exposing the freshly prepared FAI solution to ambient air for 1 min.

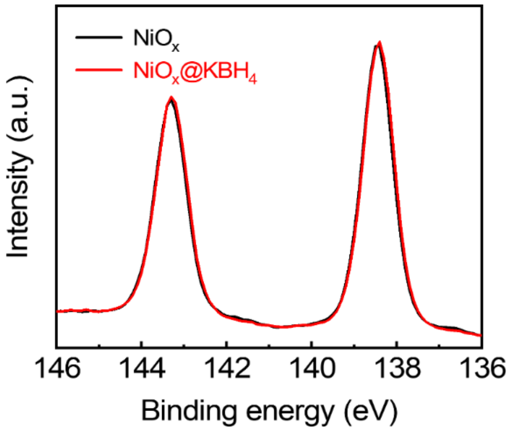

Figure S7. The XPS spectra of Pb 4f detail spectra of the perovskite films on bare NiO_x and NiO_x@KBH_4.

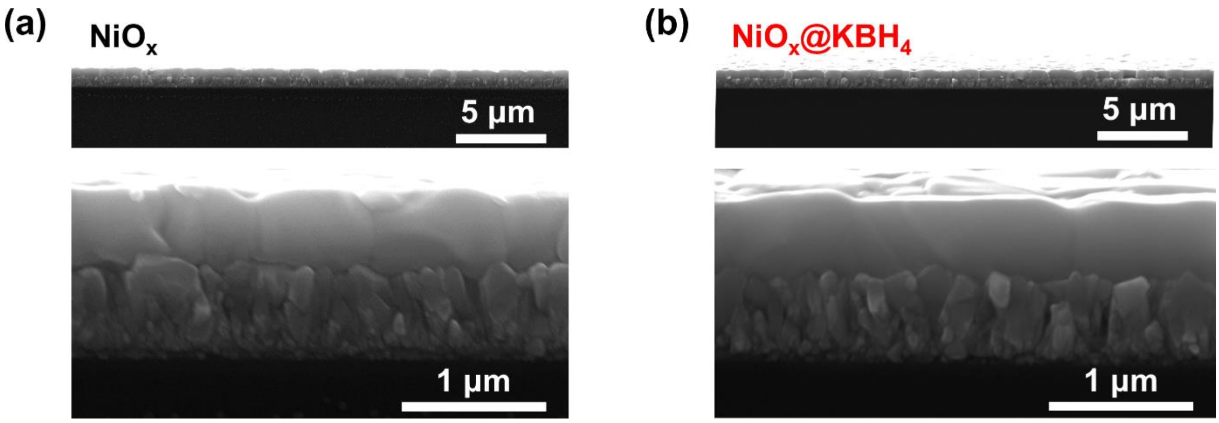

Figure S8. Cross-sectional SEM image of perovskite films on (**a**) NiO_x and (**b**) NiO_x@KBH_4, respectively.

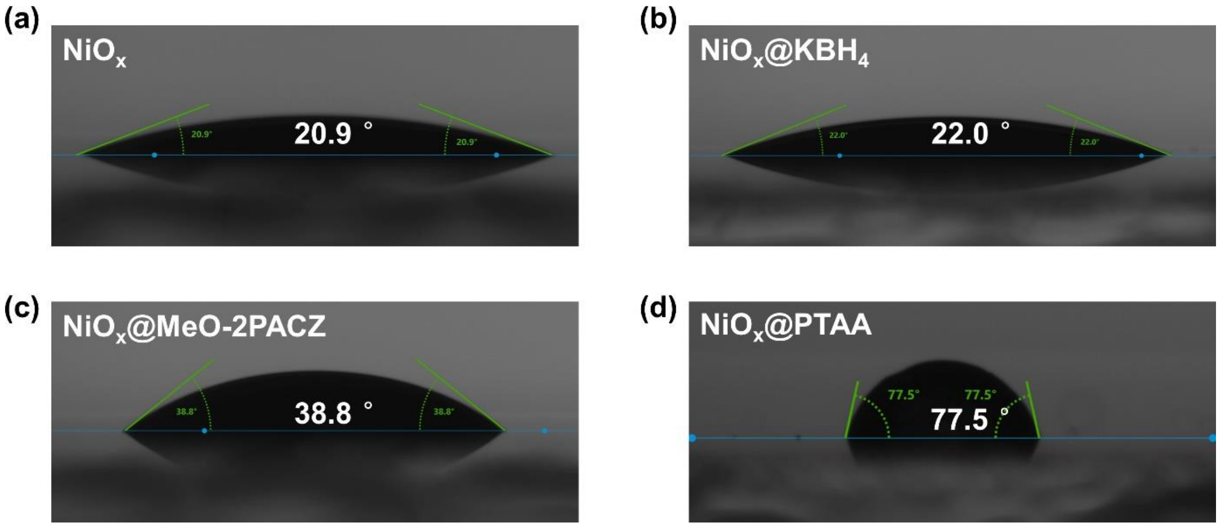

Figure S9. Surface contact angles on (**a**) NiO_x, (**b**) NiO_x@KBH_4, (**c**) NiO_x@MeO-2PACZ, and (**d**) NiO_x@PTAA.

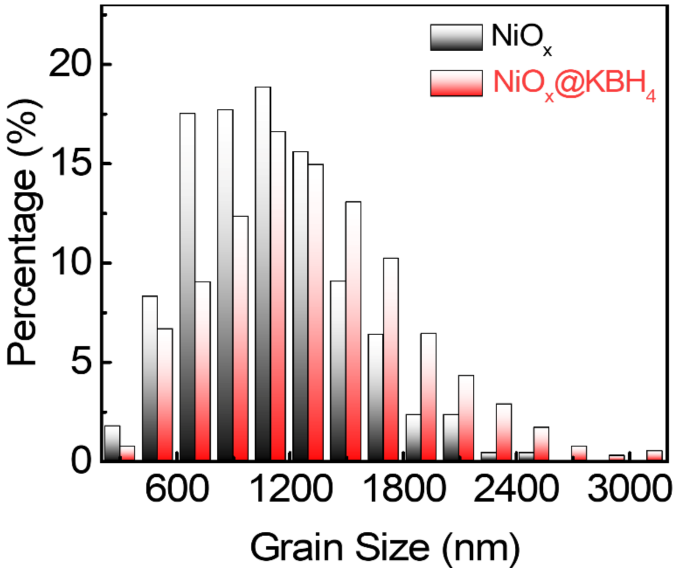

Figure S10. The grain size distribution of perovskite films deposited on NiO_x and $NiO_x@KBH_4$.

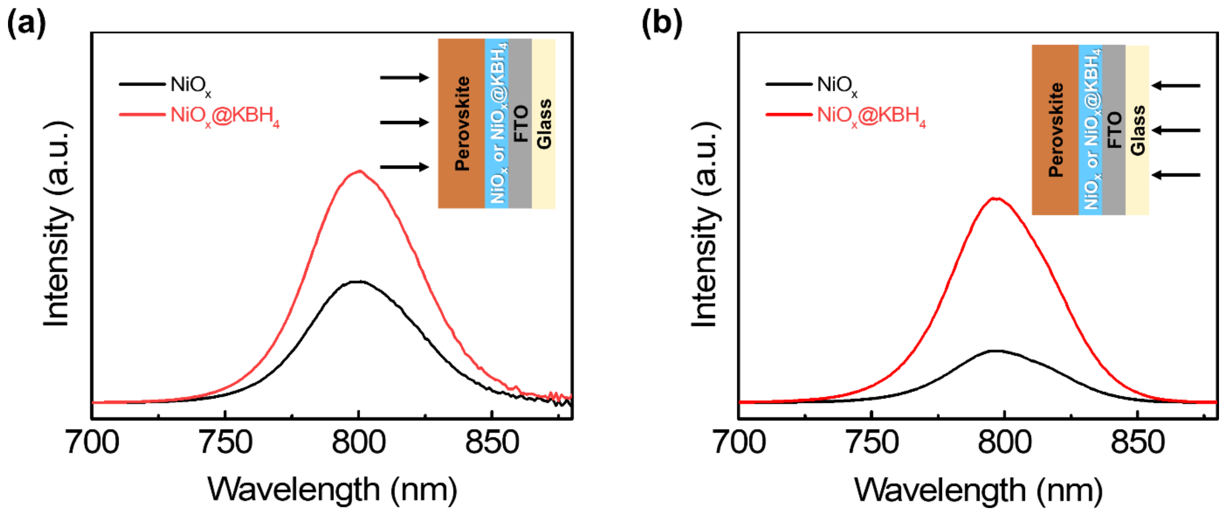

Figure S11. Steady PL spectra of perovskite films deposited on NiO_x and $NiO_x@KBH_4$, where the perovskite films were excited by a 450 nm light source (**a**) from perovskite side and (**b**) from glass substrate side.

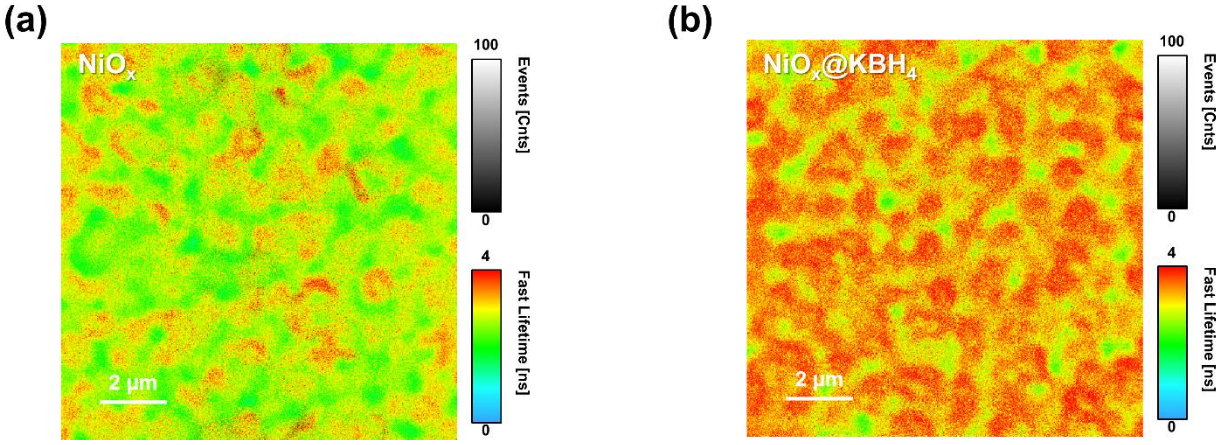

Figure S12. TRPL mapping of perovskite films deposited on (**a**) NiO_x and (**b**) NiO_x@KBH_4. Excitation from perovskite film side.

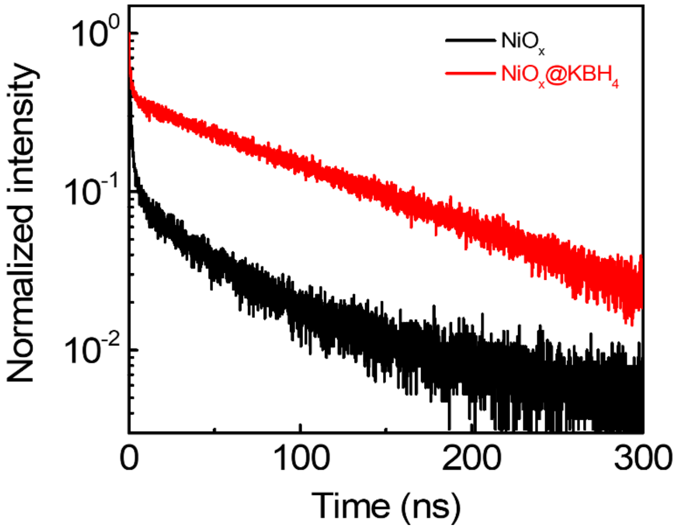

Figure S13. TRPL decay of perovskite films on NiO_x and NiO_x@KBH_4 HTLs excited from the glass side.

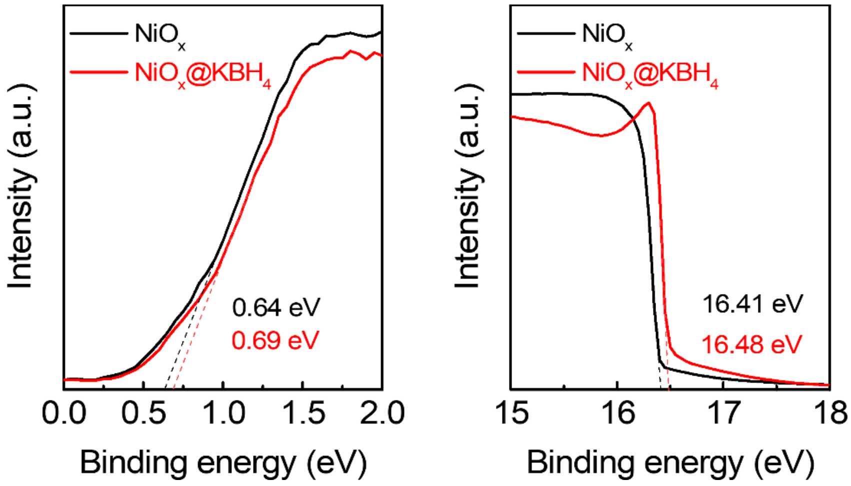

Figure S14. UPS spectra of the NiO_x and $NiO_x@KBH_4$ surface.

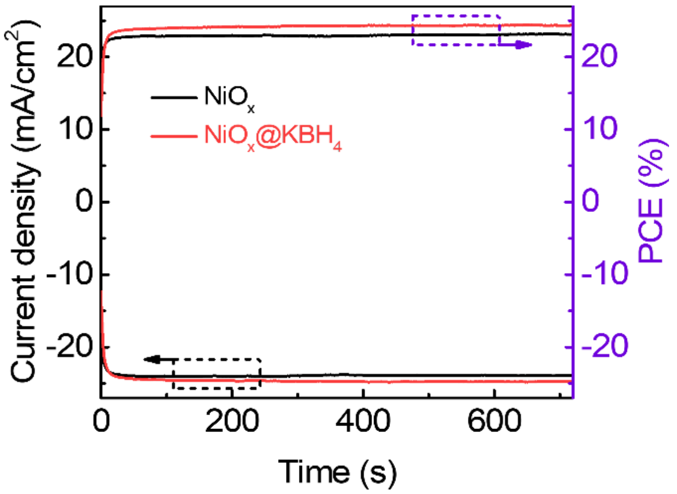

Figure S15. The stabilized power output of the perovskite solar cells fabricated on NiO_x and $NiO_x@KBH_4$ at the maximum power point for 720 s.

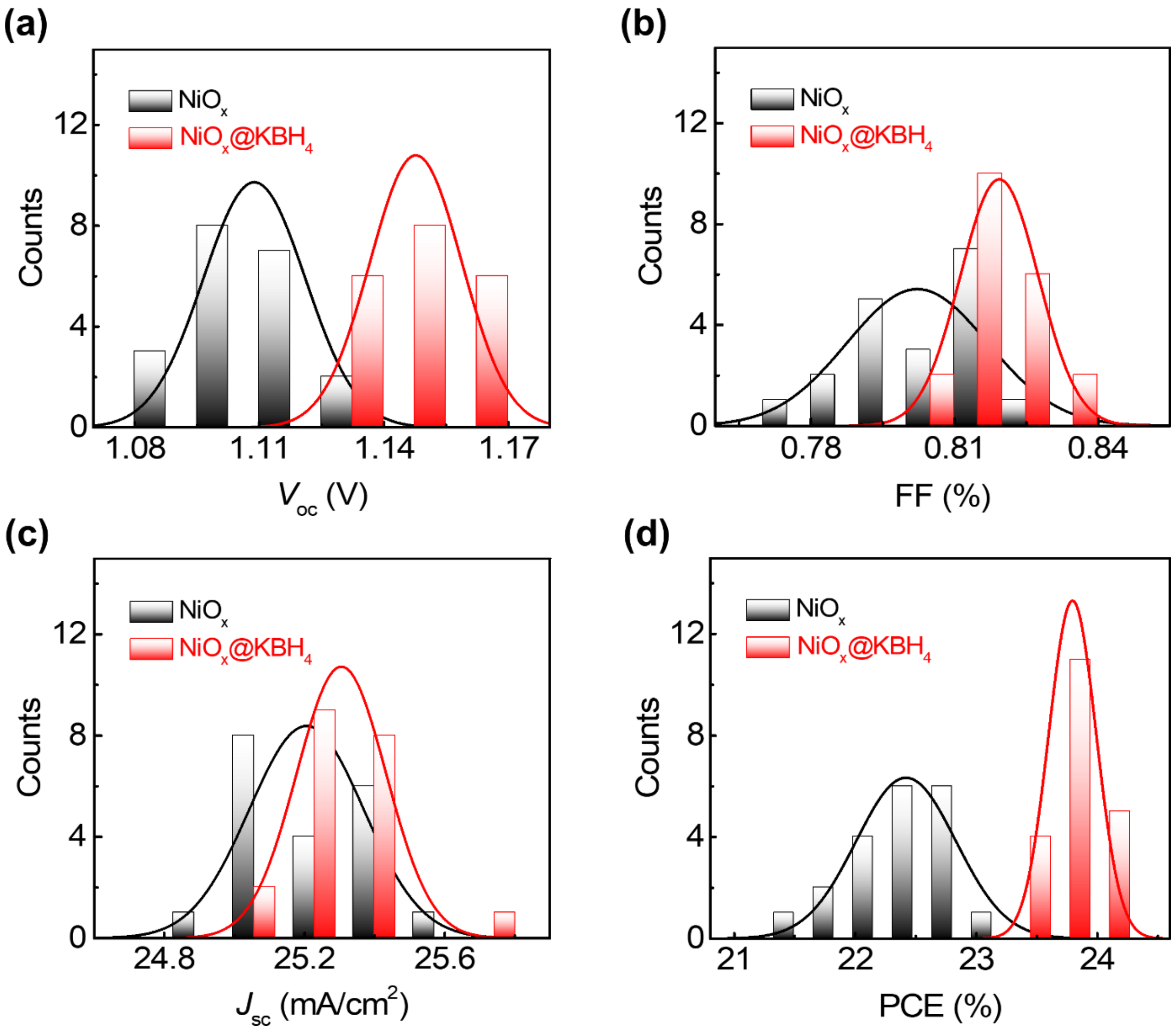

Figure S16. Photovoltaic parameter distribution of 20 PSCs deposited on NiO_x or NiO_x@KBH_4. (**a**) V_{OC}. (**b**) FF. (**c**) J_{SC}. (**d**) PCE.

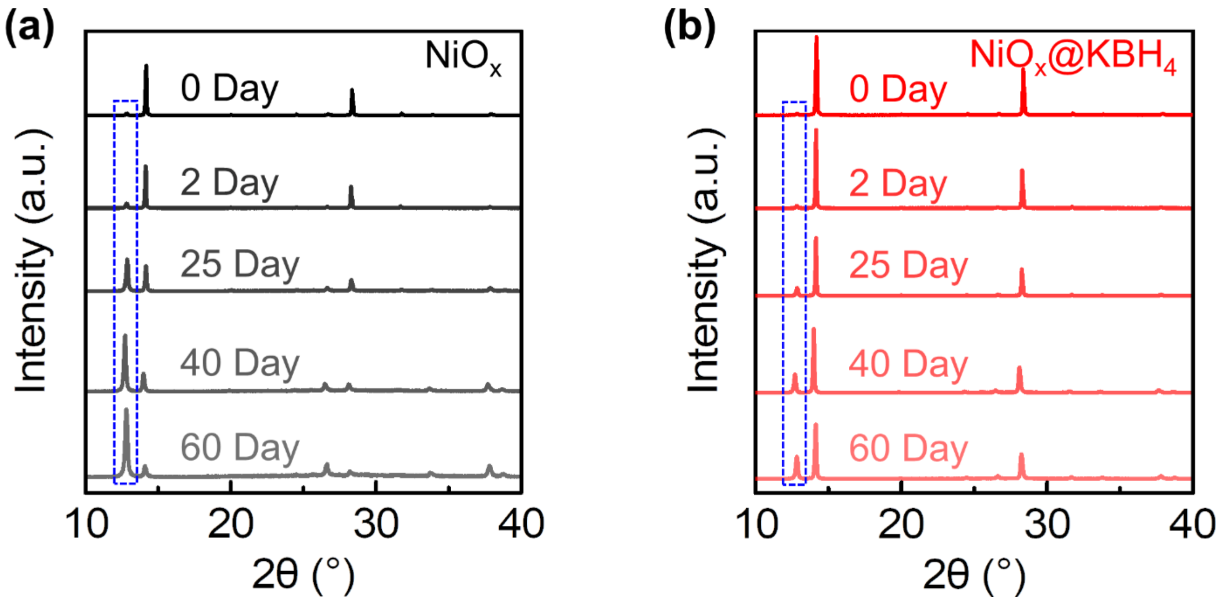

Figure S17. XRD spectra of unencapsulated perovskite films deposited on (**a**) NiO_x and (**b**) NiO_x@KBH_4 after illumination under one sun at 65 °C in N_2 atmosphere for different times.

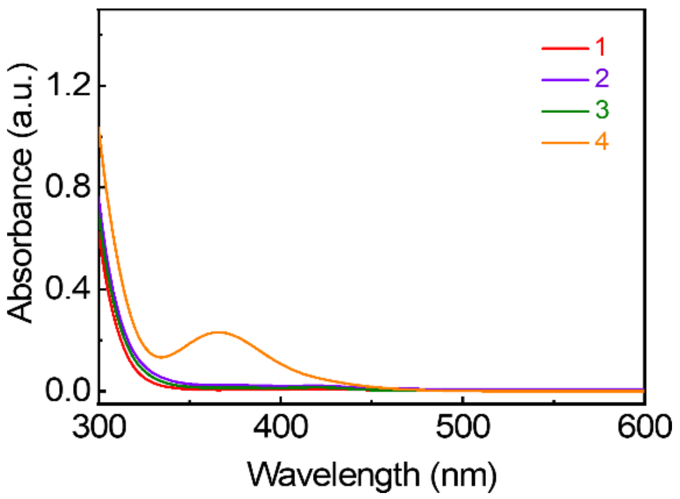

Figure S18. UV-vis absorption spectra of aged FAI solution applied to NiO_x@KBH_4 substrate for multiply cycles. The absorption spectra depict sequential cycles involving the application of 3-day-aged FAI solution onto NiO_x@ KBH_4 substrates. After each application, the solution was collected immediately back for UV-vis measurement, and then additional 3-day-aged FAI solution was applied onto the NiO_x@KBH_4 substrates to evaluate its reductive capability.

Table S1. Peak fitting parameters of the Ni 2p3/2 core level spectra of NiO_x and NiO_x@KBH_4, include binding energies and relative composition of various nickel oxidation species.

**Samples**	**NiO (eV)** **Ratio (%)**	**$Ni(OH)_2$ (eV)** **Ratio (%)**	**Ni^{3+} (eV)** **Ratio (%)**	**Ni^{4+} (eV)** **Ratio (%)**
**NiO_x**	853.62±0.04 (26.1±1.0%)	854.33±0.05 (17.2±0.8%)	855.59±0.03 (38±1.5%)	857.08±0.04 (18.7±0.3%)
**NiO_x@KBH_4**	854.64±0.07 (26.7±0.8%)	854.34±0.03 (17.2±1.5%)	855.60±0.09 (41.8±1%)	857.11±0.05 (14.3±0.1%)

Table S2. Peak fitting parameters of the Ni 2p3/2 core level spectra of NiO_x@KBH_4@FAI, include binding energies and relative composition of various nickel oxidation species.

**Samples**	**NiO (eV)** **Ratio (%)**	**$Ni(OH)_2$ (eV)** **Ratio (%)**	**Ni^{3+} (eV)** **Ratio (%)**	**Ni^{4+} (eV)** **Ratio (%)**
**NiO_x@KBH_4@FAI**	853.69 (28.2%)	854.38 (13.5%)	855.62 (44.2%)	857.15 (14.1%)

Table S3. Photovoltaic performance of perovskite solar modules in Figure 4e.

**Device**	**V_{OC} (V)**	**I_{SC} (mA)**	**FF (%)**	**PCE(%)**
NiO_x-reverse	8.64	81.60	69.50	16.9
NiO_x-forward	8.56	81.40	67.24	16.2
NiO_x@KBH_4-reverse	9.17	81.90	78.04	20.2
NiO_x@KBH_4-forward	9.16	81.77	77.93	20.1

Table S4. Comparison of PSCs in this work (red) and reported PSCs on HTL of NiO_x in the literature.

Device configuration	Area (cm^2)	V_{OC} (V)	J_{SC} (mA/cm^2)	FF (%)	PCE (%)	Ref.
ITO/NiO_x/PVK/C_{60}/CIL/Ag	0.09	1.16	23.29	81.10	21.96	[1]
ITO/NiO_x/PVK/PCBM/BCP/Ag	0.05	1.15	24.90	83.46	23.91	[2]
ITO/NiO_x/PVK/PCBM/BCP/Ag	0.09	1.14	23.44	82.80	22.13	[3]
ITO/NiO_x/PVK/PCBM/C_{60}/BCP/Ag	0.10	1.13	23.75	84.21	22.62	[4]
	1.00	1.12	22.75	79.23	20.19	
ITO/NiO_x/PVK/LiF/C_{60}/BCP/Ag	0.06	1.14	20.34	85.00	19.66	[5]
ITO/NiO_x/PVK/PCBM/BCP/Ag	0.08	1.09	23.50	75.10	19.20	[6]
FTO/NiO_x/PVK/PCBM/BCP/Ag	0.09	1.12	22.81	81.90	20.90	[7]
FTO/NiO_x/PTAA/Al_2O_3/PVK/PCBM/Ag	1.00	1.15	25.21	77.6	22.42	[8]
ITO/NiO_x/PVK/PCBM/BCP/Ag	-	1.12	22.37	81.26	20.21	[9]
ITO/NiO_x/PVK/PCBM/BCP/Ag	-	1.11	24.01	81.43	21.80	[10]
ITO/NiO_x/PVK/PCBM/BCP/Ag	0.15	1.09	22.88	77.00	19.20	[11]
ITO/NiO_x/PVK/PCBM/BCP/Ag	0.10	1.06	22.37	76.46	19.04	[12]
ITO/NiO_x/PVK/PCBM/Bphen/Ag	-	1.10	21.73	74.12	17.67	[13]
FTO/NiO_x/PVK/PCBM/Ag	0.24	1.09	19.40	80.50	17.10	[14]
ITO/NiO_x/PVK/PCBM/ZnO/Ag	0.07	1.06	22.92	70.27	17.23	[15]
	1.04	1.06	22.51	51.23	12.22	
FTO/NiO_x/PVK/PCBM/BCP/Ag	1.00	1.00	21.50	76.90	16.70	[16]
FTO/NiO_x/PVK/PCBM/ZrAcac/Ag	-	1.11	21.54	80.23	19.16	[17]

FTO/NiO_x/PVK/PCBM/ZnO/Al	0.06	1.11	22.87	70.00	17.75	[18]
ITO/NiO_x/PVK/PCBM/C_{60}/ZrAcac/Ag	0.06	1.08	22.93	81.00	19.91	[19]
FTO/NiO_x/PVK/PCBM/AgAl	-	1.09	24.27	76.55	20.31	[20]
FTO/NiO_x/PVK/PCBM/PPDIN6/Ag	0.1	1.10	23.28	79.70	20.43	[21]
ITO/NiO_x/PVK/PCBM/BCP/Ag	0.05	1.17	24.10	81.60	22.90	[22]
ITO/NiO_x/PVK/PCBM/BCP/Ag	-	1.18	24.90	81.60	24.00	[23]
FTO/NiO_x/PVK/C_{60}/BCP/Ag	0.09	1.15	24.04	85.40	23.64	[24]
FTO/NiO_x/PVK/PCBM/BCP/Ag	0.09	1.16	24.80	81.40	23.40	[25]
FTO/NiO_x/PVK/PCBM/BCP/Ag	0.06	1.16	25.19	82.44	24.17	This work

Table S5. Comparison of perovskite modules in this work (red) and reported perovskite modules on HTL of NiO_x in the literature. Abbreviation: ^{ac}, active area; ^{ap}, aperture area.

**Device configuration**	**Area (cm^2)**	**Sub-unit Cell**	***V*OC (V)**	***I*SC (mA)**	**FF (%)**	**PCE (%)**	**Ref.**
FTO/NiO_x/PVK/C_{60}/BCP/Ag	16	6	7.06	53.52	80	19.0^{ac}	[24]
	196	19	19.8	227.4	75	17.2^{ac}	
FTO/NiO_x/PVK/PCBM/BCP/Ag	178	20	20.7	198.9	78	18.6^{ac}	[25]
FTO/NiO_x/PVK/C_{60}/LiF/BCP/Bi/Ag	20.8	8	1.08	428.5	74	16.6^{ac}	[26]
ITO/NiO_x/PVK/PCBM/BCP/Ag	25	6	6.36	90.5	70	16.1^{ac}	[27]
ITO/NiO_x/PVK/PCBM/BCP/Au	10.2	5	5.27	44.9	68	15.9^{ac}	[28]
ITO/NiO_x/PVK/PCBM/BCP/Ag	35.8	10	10.8	65.7	66	14.2^{ac}	[29]

FTO/NiO_x/PVK/PCBM/BCP/Ag	29.0	8	9.17	81.90	78	20.2[ap]	This work

References

[1] W. Chen, B. Han, Q. Hu, M. Gu, Y. Zhu, W. Yang, Y. Zhou, D. Luo, F.-Z. Liu, R. Cheng, R. Zhu, S.-P. Feng, A. B. Djurišić, T. P. Russell, Z. He, *Sci. Bull.* **2021**, *66*, 991.

[2] H. Chen, S. Teale, B. Chen, Y. Hou, L. Grater, T. Zhu, K. Bertens, S. M. Park, H. R. Atapattu, Y. Gao, M. Wei, A. K. Johnston, Q. Zhou, K. Xu, D. Yu, C. Han, T. Cui, E. H. Jung, C. Zhou, W. Zhou, A. H. Proppe, S. Hoogland, F. Laquai, T. Filleter, K. R. Graham, Z. Ning, E. H. Sargent, *Nat. Photon.* **2022**, *16*, 352.

[3] P. Ru, E. Bi, Y. Zhang, Y. Wang, W. Kong, Y. Sha, W. Tang, P. Zhang, Y. Wu, W. Chen, X. Yang, H. Chen, L. Han, *Adv. Energy Mater.* **2020**, *10*, 1903487.

[4] S. Wang, Y. Li, J. Yang, T. Wang, B. Yang, Q. Cao, X. Pu, L. Etgar, J. Han, J. Zhao, X. Li, A. Hagfeldt, *Angew. Chem. Int. Ed.* **2022**, 61, e202116534.

[5] C. C. Boyd, R. C. Shallcross, T. Moot, R. Kerner, L. Bertoluzzi, A. Onno, S. Kavadiya, C. Chosy, E. J. Wolf, J. Werner, J. A. Raiford, C. de Paula, A. F. Palmstrom, Z. J. Yu, J. J. Berry, S. F. Bent, Z. C. Holman, J. M. Luther, E. L. Ratcliff, N. R. Armstrong, M. D. McGehee, *Joule* **2020**, *4*, 1759.

[6] B. Zhang, J. Su, X. Guo, L. Zhou, Z. Lin, L. Feng, J. Zhang, J. Chang, Y. Hao, *Adv. Sci.* **2020**, *7*, 1903044.

[7] S. Liu, R. Chen, X. Tian, Z. Yang, J. Zhou, F. Ren, S. Zhang, Y. Zhang, M. Guo, Y. Shen, Z. Liu, W. Chen, *Nano Energy* **2022**, *94*, 106935.

[8] Z. Wang, L. Liu, Y. Wang, Y. Ma, Z. Yang, M. Wan, H. Zhu, T. Mahmoudi, Y.-B. Hahn, Y. Mai, *Chem. Eng. J.* **2023**, *457*, 141204.

[9] J. Zhang, J. Long, Z. Huang, J. Yang, X. Li, R. Dai, W. Sheng, L. Tan, Y. Chen, *Chem. Eng. J.* **2021**, *426*, 131357.

[10] J. Zhang, J. Yang, R. Dai, W. Sheng, Y. Su, Y. Zhong, X. Li, L. Tan, Y. Chen, *Adv. Energy Mater.* **2022**, *12*, 2103674.

[11] D. Saranin, S. Pescetelli, A. Pazniak, D. Rossi, A. Liedl, A. Yakusheva, L. Luchnikov, D. Podgorny, P. Gostischev, S. Didenko, A. Tameev, D. Lizzit, M. Angelucci, R. Cimino, R. Larciprete, A. Agresti, A. Di Carlo, *Nano Energy* **2021**, *82*, 105771.

[12] Y. Lin, Y. Liao, H. Hsiao, C. Chen, *Appl. Surf. Sci.* **2020**, *504*, 144478.

[13] W. Han, G. Ren, Z. Li, M. Dong, C. Liu, W. Guo, *J. Energy Chem.* **2020**, *46*, 202.

[14] H. Yang, H. Park, B. Kim, C. Park, S. Jeong, W. Chae, W. Kim, M. Jeong, T. K. Ahn, H. Shin, *J. Phys. Chem. Lett.* **2021**, *12*, 2770.

[15] N. Kumar, H. B. Lee, S. Hwang, J.-W. Kang, *J. Mater. Chem. A* **2020**, *8*, 3357.

[16] E. Ochoa-Martinez, S. Bijani-Chiquero, M. D. V. Martínez De Yuso, S. Sarkar, H. Diaz-Perez, R. Mejia-Castellanos, F. Eickemeyer, M. Grätzel, U. Steiner, J. V. Milić, *Adv. Sci.* **2023**, *10*, 2302549.

[17] X. Yin, J. Zhai, P. Du, N. Li, L. Song, J. Xiong, F. Ko, *Chemsuschem* **2020**, *13*, 1006.

[18] U. K. Thakur, P. Kumar, S. Gusarov, A. E. Kobryn, S. Riddell, A. Goswami, K. M. Alam, S. Savela, P. Kar, T. Thundat, A. Meldrum, K. Shankar, *ACS Appl. Mater. Interfaces* **2020**, *12*, 11467.

[19] D. Ouyang, C. Chen, Z. Huang, L. Zhu, Y. Yan, W. C. H. Choy, *ACS Appl. Mater. Interfaces* **2021**, *13*, 16611.

[20] Y. Liu, J. Duan, J. Zhang, S. Huang, W. Ou-Yang, *ACS Appl. Mater. Interfaces* **2020**, *12*, 1, 771.

[21] C. Hu, Y. Bai, S. Xiao, K. Tao, W. K. Ng, K. S. Wong, S. H. Cheung, S. K. So, Q. Chen, S. Yang, *Sol. RRL* **2020**, *4*, 2000270.

[22] B. Chen, H. Chen, Y. Hou, J. Xu, S. Teale, K. Bertens, H. Chen, A. Proppe, Q. Zhou, D. Yu, K. Xu, M. Vafaie, Y. Liu, Y. Dong, E. H. Jung, C. Zheng, T. Zhu, Z. Ning, E. H. Sargent, *Adv. Mater.* **2021**, *33*, 2103394.

[23] H. Wang, W. Zhang, B. Wang, Z. Yan, C. Chen, Y. Hua, T. Wu, L. Wang, H. Xu, M. Cheng, *Nano Energy* **2023**, *111*, 108363.

[24] C. Li, Y. Zhang, X. Zhang, P. Zhang, X. Yang, H. Chen, *Adv. Funct. Mater.* **2023**, 2214774.

[25] M. Du, S. Zhao, L. Duan, Y. Cao, H. Wang, Y. Sun, L. Wang, X. Zhu, J. Feng, L. Liu, X. Jiang, Q. Dong, Y. Shi, K. Wang, S. (Frank) Liu, *Joule* **2022**, *6*, 1931.

[26] Z. Yang, W. Zhang, S. Wu, H. Zhu, Z. Liu, Z. Liu, Z. Jiang, R. Chen, J. Zhou, Q. Lu, Z. Xiao, L. Shi, H. Chen, L. K. Ono, S. Zhang, Y. Zhang, Y. Qi, L. Han, W. Chen, *Sci. Adv.* **2021**, *7*, eabg3749.

[27] Z. Huang, X. Hu, Z. Zhao, X. Meng, M. Su, T. Xue, J. Chi, H. Xie, Z. Cai, Y. Chen, L. Li, Y. Song, *Adv. Energy Mater.* **2021**, 11, 2101291.

[28] F. D. Giacomo , L. A. Castriotta, F. U. Kosasih, D. D. Girolamo,C. Ducati and A. D. Carlo, *Micromachines* **2020**, *11*, 1127.

[29] E. Bi, W. Tang, H. Chen, Y. Wang, J. Barbaud, T. Wu, W. Kong, P. Tu, H. Zhu, X. Zeng, J. He, S. Kan, X. Yang, M. Grätzel, L. Han, *Joule* **2019**, *3*, 2748.
